# Advances in exercise to alleviate sarcopenia in older adults by improving mitochondrial dysfunction

**DOI:** 10.3389/fphys.2023.1196426

**Published:** 2023-07-05

**Authors:** Yang Zhu, Xuchang Zhou, Aiyuan Zhu, Shijing Xiong, Jun Xie, Zhenmin Bai

**Affiliations:** School of Sports Medicine and Rehabilitation, Beijing Sport University, Beijing, China

**Keywords:** sarcopenia, exercise, mitochondrial dysfunction, mitochondrial biogenesis, mitochondrial apoptosis

## Abstract

Sarcopenia is a chronic degenerative disease affecting primarily older adults. A growing aging population is gradually increasing the number of patients suffering from sarcopenia, placing increasing financial pressure on patients’ families and society in general. There is a strong link between mitochondrial dysfunction and sarcopenia pathogenesis. As a result, treating sarcopenia by improving mitochondrial dysfunction is an effective strategy. Numerous studies have demonstrated that exercise has a positive effect on mitochondrial dysfunction when treating sarcopenia. Exercise promotes mitochondrial biogenesis and mitochondrial fusion/division to add new mitochondria or improve dysfunctional mitochondria while maintaining mitochondrial calcium homeostasis, mitochondrial antioxidant defense system, and mitochondrial autophagy to promote normal mitochondrial function. Furthermore, exercise can reduce mitochondrial damage caused by aging by inhibiting mitochondrial oxidative stress, mitochondrial DNA damage, and mitochondrial apoptosis. Exercise effectiveness depends on several factors, including exercise duration, exercise intensity, and exercise form. Therefore, Moderate-intensity exercise over 4 weeks potentially mitigates sarcopenia in older adults by ameliorating mitochondrial dysfunction. HIIT has demonstrated potential as a viable approach to addressing sarcopenia in aged rats. However, further investigation is required to validate its efficacy in treating sarcopenia in older adults.

## Introduction

Sarcopenia is characterized by a progressive decline in skeletal muscle strength, mass, and function. It is also a progressive and generalized degenerative disease of aging (Cruz-Jentoft and Sayer, 2019). Sarcopenia is characterized by symptoms such as weakness, loss of muscle strength, and loss of muscle mass, which are affected by several factors, including age, gender, physical activity, and chronic illness (Wu et al., 2022). Sarcopenia may worsen symptoms of chronic diseases such as chronic obstructive pulmonary disease, chronic heart failure, diabetes, and osteoporosis. In addition, it can cause motor dysfunction in these patients, increasing the risk of falls, fractures, and even the loss of independence. Global aging and sedentary lifestyles have led to an increased prevalence of sarcopenia (de Rezende et al., 2014; Chen Z. et al., 2021). A meta-analysis found that 10%–27% of people globally suffer from the condition (Petermann-Rocha et al., 2022). Therefore, sarcopenia has become a serious health concern. Moreover, Sarcopenia places a tremendous financial burden on the patient’s family and society as a whole. The current systematic review indicates that exercise can significantly improve muscle strength and physical performance in older adults with sarcopenia, but does not alter muscle mass (Escriche-Escuder et al., 2021; Wang H. et al., 2022). This may be due to the close connection between muscle strength and physical performance (Kim YH. et al., 2016). Resistance exercise, aerobic exercise, and mixed exercise are common exercise forms for treating sarcopenia (Chen N. et al., 2021; Talar et al., 2021; Huang et al., 2022; Shen et al., 2023). However, resistance and mixed exercise are generally recognized as the most effective intervention for increasing muscle mass (Lu et al., 2021; Shen et al., 2023). Additionally, HIIT, blood flow restriction training, and vibration exercise may be effective strategies for improving muscle strength and physical performance in older adults with sarcopenia (Wu et al., 2020; Hayes et al., 2021; Lu et al., 2021; Alizadeh Pahlavani, 2022; Liu et al., 2022). A large-scale clinical trial will be required in the future to verify the efficacy of HIIT, blood flow restriction training, and vibration training.

Besides improving muscle mass, strength, and physical performance, exercise for sarcopenia causes adaptive changes in mitochondrial function as well. Mitochondria are the most critical organelles for energy production, metabolism of free radicals, and programmed apoptosis of cells. The mitochondrial quality control system protects mitochondrial quantity and quality through the regulation of mitochondrial biosynthesis, mitochondrial dynamic homeostasis, and mitochondrial autophagy (Stotland and Gottlieb, 2015). In this way, mitochondrial dysfunction caused by aging can be alleviated. Therefore, previous studies provided preliminary evidence that dysfunctional mitochondrial quality control may contribute to muscle atrophy and physical disability associated with aging (Joseph et al., 2012). Moreover, increased reactive oxygen species (ROS) in older patients with sarcopenia can disrupt the balance between oxidative stress and antioxidant defenses in mitochondria. As a result, mitochondrial apoptosis and damage to mitochondrial DNA (mtDNA) are increased, mitochondrial calcium (Ca^2+^) homeostasis is disrupted, and mitochondrial fusion/fission and mitochondrial biogenesis are inhibited (Chabi et al., 2008; Andersson et al., 2011; Alway et al., 2017). Therefore, mitochondrial dysfunction not only results from aging but also plays a crucial role in the pathogenesis of Sarcopenia. However, no studies have confirmed that pharmacological therapies can restore mitochondrial function in aging skeletal muscles. Exercise improves mitochondrial dysfunction and prevents progressive loss of muscle function and mass with age (Carter et al., 2015). Therefore, exercise is an effective strategy for improving muscle function and quality of life in older adults by stimulating mitochondrial adaptation. Nevertheless, it is unclear what form, frequency, and duration of exercise can be prescribed for the treatment of sarcopenia. The purpose of this paper is to review the research literature on exercise for improving mitochondrial dysfunction in the treatment of sarcopenia in recent years and summarize the mechanisms by which exercise regulates sarcopenia through mitochondria, to provide a theoretical foundation and references for studies regarding optimal exercise recommendations for alleviating sarcopenia.

## Diagnosis of sarcopenia

The concept of sarcopenia is constantly evolving (Dent et al., 2021). Sarcopenia was initially understood to be a decrease in muscle strength (Langer et al., 2019). As clinical medicine has developed, the European Working Group on Sarcopenia in Old People (EWGSOP) introduced a contemporary definition of sarcopenia in 2010, which includes low muscle mass, low muscle strength, and/or low physical performance (Cruz-Jentoft et al., 2010). Currently, there is no gold standard for diagnosing sarcopenia. However, sarcopenia can also be diagnosed by low muscle mass, low muscle strength, and low physical performance. Recently, EWGSOP (Cruz-Jentoft et al., 2019), the Asian Working Group for Sarcopenia (AWGS) (Chen et al., 2020), and the Sarcopenia Definition and Outputs Consortium (SDOC) (Bhasin et al., 2020) have updated the diagnostic consensus for sarcopenia (Table 1). These consensus guidelines differ in their diagnostic criteria for sarcopenia. EWGSOP and AWGS evaluation content are similar, but critical values differ. The two consensuses may target different ethnic and geographical groups (Woo et al., 2014). The SDOC excludes muscle mass from sarcopenia diagnostic criteria, perhaps due to its inability to accurately predict adverse health outcomes (Bhasin et al., 2020). Consequently, it will be necessary to develop a universally applicable diagnostic method for sarcopenia in the future.

**TABLE 1:** Clinical consensus comparison of the main diagnostic tools for sarcopenia in older adults.

Name year	Muscle strength	Muscle Mass	Physical performance	Diagnosis
EWGSOP 2019 Cruz-Jentoft et al. (2010)	Grip strength: M: < 27 kg F: <16 kg	DXA ASM: M:< 20 kg F:< 15 kg	Gait speed: ≤ 0.8 m/s	Sarcopenia = Low Muscle Mass + Low Muscle Strength OR Low Physical Performance
	Chair stand: >15 s for five rises	ASM/height^2^ M:< 7.0 kg/m^2^ F: < 5.5 kg/m^2^	SPPB: ≤ 8 point score	Severe Sarcopenia = Low Muscle Mass + Low Muscle Strength + Low Physical Performance
			TUG: ≥ 20 s 400 m walk test: Non-completion or ≥6 min for completion	
AWGS 2020 Cruz-Jentoft et al. (2019)	Handgrip strength: M: < 28 kg F: < 18 kg	DXA-ASM: M: < 7.0 kg/m^2^ F: < 5.4 kg/m^2^	6-m walk: < 1 m/s	Probably Sarcopenia = Low Muscle Strength
		BIA-ASM: M: < 7.0 kg/m^2^ F: < 5.7 kg/m^2^	5-time chair stand test: ≥ 12 m/s	Sarcopenia = Low Muscle Mass OR Low Muscle Strength
			SPPB: ≤ 9 point score	Severe Sarcopenia = Low Muscle Mass + Low Muscle Strength + Low Physical Performance
SDOC 2020 Chen et al. (2020)	Handgrip strength (maximal): M: < 35.5 kg F: < 20 kg		Gait Speed (usual): M:> 0.8 m/s F: >0.8 m/s	Sarcopenia = Low Grip Strength and Low usual Gait Speed

EWGSOP, the European Working Group on Sarcopenia in Older People; DXA, Dual-energy X-ray absorptiometry; ASM, appendicular skeletal muscle mass; SPPB, short physical performance battery; AWGS, asian working group for sarcopenia; TUG, time up and go test; BIA, bioelectrical impedance analysis; SDOC, sarcopenia definition and outcomes consortium; F, female; M, male.

### Exercise ameliorates sarcopenia by increasing mitochondrial biogenesis

Mitochondrial biogenesis is the process of intracellular production of newly formed and functioning normal mitochondria (Johnson et al., 2021). Some proteins play a significant role in mitochondrial biogenesis, but their expression decreases with age (Carter et al., 2018). PGC-1α plays a major role in regulating mitochondrial biogenesis in muscle cells and is positively associated with mitochondrial biogenesis. Numerous studies have confirmed that PGC-1α is regulated by AMP-activated protein kinase (AMPK), silence information regulator 1 (SIRT1), and p53 (Zong et al., 2002; Saleem et al., 2009; Menzies et al., 2013). Under stress conditions such as exercise, PGC-1α controls the expression of multiple transcription factors (nuclear respiratory factors-1 (NRF-1) and nuclear respiratory factors-1 (NRF-2) and the expression of mitochondrial transcription factor A (TFAM) to produce newly formed mitochondria (Scarpulla et al., 2012; Granata et al., 2018). In addition, the expression of PGC-1α in older muscles is decreased as a result of a decrease in upstream signaling (Ljubicic and Hood, 2009; Carter et al., 2018). Therefore, PGC-1α expression cannot be ignored when researching the effects of exercise on mitochondrial biogenesis in sarcopenic patients. But there are also studies suggesting that PGC-1α、P53 is not necessary for adaptive response to exercise training (Rowe et al., 2012; Beyfuss et al., 2018). Even older skeletal muscles without PGC-1α and P53 can adapt to exercise to maintain normal metabolic functions (Table 2).

**TABLE 2:** An overview of studies on exercise’s effects on mitochondrial biogenesis.

Author year	Subjects	Exercise forms	Exercise duration	Exercise intensity	Protein level	Change
Kitaoka 2015 Beyfuss et al. (2018)	rat	Resistance exercise	4 weeks, 3 day/week			no change
Mesquita 2020 Kitaoka et al. (2015)	older adults	resistance training	10 weeks, 2 day/week		PGC-1α, TFAM,NRF1	no change
Koltai 2012 Mesquita et al. (2020)	rat	treadmill running	6 weeks, 7 days/week	60% Vo_2_max	PGC1α,SIRT1,TFAM,NRF1	Increase
Kang 2013 Koltai et al. (2012)	rat	treadmill running	12 weeks, 5 day/week	Moderate strength	PGC-1α, SIRT1,AMPK	Increase
Moore 2019 Kang et al. (2013)	mice	treadmill running	90 mint	Moderate strength	PGC-1α, TFAM,NRF-1	Increase
Gao 2021 Moore et al. (2019)	rats	treadmill running	8 months	Moderate strength	AMPK, PGC-1α	Increase
Han 2022 Gao et al. (2021)	rats	treadmill running	8 months, 5 days/week	MICT:75%–80% O_2_max HIIT: 45%–55% + 85–95% O_2max_	SIRT3, PGC-1α	Increase
Pirani 2023 Han et al. (2022)	old rats	treadmill running	12 weeks HIIT:16-18 min MICT:30-60 mim	MICT: 65%–70% VO_2max_ HIIT:85%–90% + 45%-75%VO_2max_	AMPK, PGC-1α,ERR	Increase
Heather 2018 (Johnson et al., 2021)	rats	Acute contractile activity			NRF-2, PGC-1α	Increase
Edgett 2013 Pirani et al. (2023)	adults	cycle ergometer	3 h	the peak work rate of 100% or 73% or 133%	PGC-1α	Increase
Granata 2016 Edgett et al. (2013)	adults	cycle	4 weeks, 3 days/week	200% or 90% or 65% of peak power output	PGC-1α, p53	Increase
Granata 2020 Granata et al. (2016)	adults	electronically braked cycle ergometer	20 days, 2 times/day	HVT	PGC-1α, p53	Increase

Exercise form plays a critical role in mitochondrial biogenesis (Table 2). Kitaoka found that a 4-week resistance exercise had little effect on mitochondrial biogenesis in a somatic animal model (Kitaoka et al., 2015). Further, Mesquita investigated the histopathology of the lateral femoral muscle in older adult subjects and concluded that 10-week resistance exercise did not affect mitochondrial biogenesis as well (Mesquita et al., 2020). Therefore, resistance exercise does not affect mitochondrial biogenesis. However, 6 weeks of endurance exercise can reverse the decline in SIRT1, AMPK, and PGC-1α in aged rats, and diminish the gap in mitochondrial biogenesis-related proteins between aged and young rats (Koltai et al., 2012). Aged rats undergoing endurance exercise for 12 weeks exhibited significantly higher levels of PGC-1α, TFAM, SIRT1, and AMPK than other sedentary rats (Kang et al., 2013). Therefore, upregulation of PGC-1α signaling may be responsible for promoting mitochondrial biogenesis in aged rat skeletal muscle after endurance training. This conclusion has also been confirmed by human experiments. Endurance exercise can increase mitochondrial biogenesis by increasing the expression of mitochondrial biogenesis-related proteins (e.g., PGC-1α, TFAM, NRF-1), as well as activating the AMPK/PGC-1Α signaling pathway (Moore et al., 2019; Gao et al., 2021). Therefore, endurance training promotes mitochondrial biogenesis and is correlated with the duration of endurance training. However, research has shown that HIIT promotes mitochondrial biogenesis more effectively than moderate-intensity continuous training (MICT) (Han et al., 2022; Pirani et al., 2023). Therefore, HIIT has demonstrated superior efficacy in augmenting mitochondrial biogenesis in aged rats with sarcopenia.

Exercise increases PGC-1α expression in older adults, pointing to the possibility that older muscles can respond to exercise efficiently to increase mitochondrial biogenesis (Carter et al., 2018). In addition, it was confirmed that PGC-1α and p53 protein levels increased with training volumes, and a short-term decrease in training volumes decreased the levels of both proteins, indicating that training volume remains a factor influencing training-induced mitochondrial biogenesis (Granata et al., 2018). Nonetheless, the upregulation of PGC-1α does not exhibit a linear correlation with escalating training volumes. Edgett’s experimental findings indicate that HIIT with 100% peak aerobic power is the most effective in promoting PGC-1α expression following a single exercise session of varying intensities (Edgett et al., 2013). While the 73% and 133% conditions have comparable effects on mitochondrial biogenesis, both fall slightly short of the biogenesis observed under 100% conditions (Edgett et al., 2013). Furthermore, the activation of PGC-1α transcription by AMPK may not be influenced by exercise intensity and may not be augmented by ultra-high-intensity exercise (Edgett et al., 2013). Moreover, it has been observed that as the level of physical exertion escalates, the manifestation of PGC-1α is inferior to that of Electromyography derived muscle activation, potentially leading to a dampening of PGC-1α signaling mediated activation. Consequently, it remains uncertain whether a single bout of supramaximal intensity training can confer exercise advantages that surpass those attained through submaximal and maximal intensity exercises. Conversely, Granada has demonstrated that the intensity-dependent regulation of PGC-1α protein may persist beyond the pinnacle of power after prolonged durations and intensities of exercise (Granata et al., 2016). The inconsistent timing of muscle biopsy, exercise duration, and exercise frequency in the two experiments may have contributed to the observed possibility. However, Granata confirmed this conclusion with guaranteed consistent muscle biopsy timing (Granata et al., 2020). These findings underscore the significance of exercise intensity in mitochondrial biogenesis. While optimal exercise intensity remains a topic of debate, both endurance training and HIIT have been shown to promote mitochondrial biogenesis. For older adults, endurance training is recommended based on their physical condition.

### Exercise ameliorates sarcopenia by balancing mitochondrial dynamics

Mitochondrial dynamics include mitochondrial fusion and fission. Mitochondrial autophagy, aging, and decreased physical activity promote mitochondrial fragmentation, which leads to mitochondrial dysfunction (Wai and Langer, 2016). Mitochondrial fission isolates dysfunctional mitochondria, improving mitochondrial function (Wai and Langer, 2016). Additionally, mitochondrial fusion plays a significant role in maintaining mitochondrial function. For example, mitochondrial fusion regulates oxidative phosphorylation (OXPHOS) and mitochondrial DNA replication (Silva Ramos et al., 2019). Mitochondrial fusion processes include mitofusin 1 (Mfn1) and mitofusin 2 (Mfn2) mediated mitochondrial outer membrane fusion and optical atrophy protein 1 (Opa1) mediated mitochondrial inner membrane fusion (Bell et al., 2019). Mahmoodzadeh (Mahmoodzadeh et al., 2021) also confirmed that Ahnak1 is likely located in the outer mitochondrial membrane and plays an important role in mitochondrial fusion. The mitochondrial division is mediated by mitochondrial fusion protein 1 (FIS1), dynamin-related protein 1 (Drp1), and mitochondrial fission factor (MFF) (Liu et al., 2021). In contrast, to direct mitochondrial fission, there has been evidence that FIS1 inhibits mitochondrial fusion (Yu et al., 2019). The downregulation of Mfn2, FIS1, Drp1, and Opa1 in sarcopenia in old people can be associated with a significant decline in exercise performance (Liu et al., 2021). This suggests that aging and exercise can alter the dynamic balance of mitochondria. Therefore, the mitochondrial dynamic imbalance is associated with sarcopenia and skeletal muscle atrophy.

Observations of mitochondria in skeletal muscles after exercise indicate that they exist in a more fused state (Mishra et al., 2015). But acute exercise does not alter mitochondrial fusion proteins expression. For example, Picard found that after a voluntary round run of 3 hours on mice, mitochondrial morphology and Mfn2 and Opa1 abundance did not change (Picard et al., 2013). Acute resistance exercise in rats has also verified this conclusion (Kitaoka et al., 2015). Moreover, Marshall demonstrated in human experiments that acute resistance exercise may not alter mitochondrial function in an older adult with muscle disuse (Marshall et al., 2022). However, research has shown that prolonged exercise training may result in mitochondrial fusion. Resistance training for 4 weeks increased the levels of Mfn1, Mfn2, and Opa1 proteins in the gastrocnemius muscles of rats (Kitaoka et al., 2015). Nevertheless, this only increased mitochondrial bulk density, regardless of mitochondrial content. Furthermore, resistance training for 10 weeks can also increase the levels of Mfn1, Mfn2, and Opa1 proteins in older people (Mesquita et al., 2020). Aerobic exercise for 20 weeks can increase the levels of OPA1 and MFF in the gastrocnemius muscle of healthy adult males (Zoladz et al., 2022). The use of HIIT and MICT for 12 weeks has been shown to reduce Drp1 and FIS1 levels and to increase the levels of Mfn1, Mfn2, and Opa1 to promote mitochondrial fusion (Li B. et al., 2019). However, Wyckelsma believes that HIIT is more effective (Wyckelsma et al., 2017). As a result of the correlation between exercise intensity and Mfn2 expression (Fiorenza et al., 2018). While the majority of studies have been conducted on healthy adults, the number of mitochondria in the muscles of young and healthy older adults is similar (Wyckelsma et al., 2017). Therefore, chronic exercise for 4 weeks or more reduces sarcopenia by activating mitochondrial fusion.

FIS1 and Drp1 protein levels in the mitochondria of the gastrocnemius muscle did not change within 1 h following acute resistance exercise (Kitaoka et al., 2015). However, Moore developed a mouse model (mDrp1) which maintained high levels of FIS1 and Opa1 for 3 h following the cessation of endurance training (Moore et al., 2019). These results demonstrate that exercise form and Drp1 signal transduction play a significant role in regulating exercise performance and adapting to endurance training. In contrast, acute sprint interval training of different intensities can increase DRP1 levels in the lateral femoral muscle (Granata et al., 2017). DRP1 expression is influenced by the impact of high-intensity exercise on β-adrenergic stimulation of adrenaline (Fiorenza et al., 2018). Moreover, Ding found that the levels of FIS1 mRNA and protein increased significantly after 120–150 min of endurance training (Ding et al., 2010). The rapid expression of the FIS1 gene in skeletal muscle may be a result of increased metabolic demands during exercise. This may have a significant impact on the efficiency of oxidative phosphorylation. Therefore, exercise can improve mitochondrial dynamics in older adults with sarcopenia. Long-term, moderately intense, or highly intense exercise can reduce sarcopenia and muscle atrophy in older adults.

### Exercise ameliorates sarcopenia by inducing mitochondrial autophagy

The mitochondria within the cell undergo depolarization due to external stimuli such as ROS, a lack of nutrients, and the aging of the cells. Mitochondrial autophagy mechanisms are usually divided into two categories: ubiquitin-dependent pathways and non-ubiquitin-dependent pathways (Lu et al., 2023). The ubiquitin-dependent pathway refers to the widespread ubiquitination of mitochondrial surface proteins that promote mitochondrial autophagy. The PTEN-induced oxidative kinase protein 1 (PINK1)/Parkin pathway is currently the most extensively studied pathway (Guan et al., 2018). When mitochondrial membrane potential (MMP, ΔΨ m) When damaged, the pathway of PINK1 entering the inner mitochondrial membrane is blocked, leading to the stable aggregation of PINK1 on the cytoplasmic surface of the outer membrane of mitochondria. In parallel, this will recruit and activate Parkin, and the spatial conformation of Parkin protease will change into activated E3 ubiquitin ligase, which will ubiquitinate proteins in mitochondria (Riley et al., 2013). In addition, PINK1 directs the recruitment of autophagic receptor proteins (such as Nip3-like protein X (NIX), BCL2-interacting protein 3(BNIP3), and FUN14 domain containing 1(FUNDC1)) to mitochondria through ubiquitin phosphorylation (Lu et al., 2023). The receptor proteins recruit LC3, which enables autophagosomes to engulf mitochondria. Moreover, the expression of LC3, BNIP3, beclin1, Atg7, p62, and Parkin decreases with age (Liang et al., 2020). Therefore, autophagy levels are low in the skeletal muscles of older animals. Under stress factors, such as exercise, AMPK promotes autophagy by promoting mitochondrial division (Zhang and Lin, 2016).

A cross-sectional study has shown that older adults who exercise have higher levels of mitochondrial phagocytosis proteins Parkin and BNIP3 (Dethlefsen et al., 2018; Balan et al., 2019). Therefore, an active lifestyle contributes to mitochondrial autophagy. Nevertheless, mitochondrial autophagy varies according to the age at which exercise is initiated. As compared with mice aged 18 months, mice aged 8 months had increased mitochondrial autophagy activity, improving their physical function (Gao et al., 2020). Therefore, older adults should begin training as early as possible and maintain their exercise habits to effectively prevent sarcopenia. Acute exercise such as downhill running acutely, swimming, or running on a treadmill can promote the expression of mitochondrial autophagy-related proteins (Kim et al., 2012; Lenhare et al., 2017; Shang et al., 2019). Moreover, chronic exercises such as endurance training, downhill/uphill training, and resistance training increased autophagic activity in the gastrocnemius muscle of aged rats (Kim et al., 2013; Kim JS. et al., 2016; Zeng et al., 2020). Therefore, exercise duration, exercise form, and exercise intensity are associated with differences in autophagy marker expression. Meta-analyses have shown that long-term aerobic and resistance exercise may increase the expression of autophagy-related proteins in aging skeletal muscles (Wang C. et al., 2022). However, a prolonged period of high-intensity exercise may lead to excessive autophagy, while a short period of low-intensity exercise (intervention duration< 12 weeks, frequency< 3 times/week) may not reach the level of exercise-induced autophagy that is required for metabolic adaptation. This conclusion was also supported by Yu’s comparative experiment (Yu et al., 2020). Furthermore, Yu found that exercise can increase the LC3-II/LC3-I ratio as well as decrease the expression of the p62 protein (Yu et al., 2020). But under the same exercise intensity, the autophagy activity of skeletal muscle mitochondria after 4 weeks of exercise is higher than that after 2 weeks of exercise (Yu et al., 2020). Under the same exercise time, the mitochondrial autophagy activity of high-intensity exercise was higher than that of moderate-intensity exercise (Yu et al., 2020). Therefore, different exercise durations and intensities have varying effects on autophagy-related protein expression. However, Yu’s experimental time was relatively short, and the results of the meta-analysis may be heterogeneous. Nevertheless, moderate-intensity exercise for more than 4 weeks increases mitochondrial autophagy.

### Exercise ameliorates sarcopenia by improving mitochondrial Ca^2+^ homeostasis

The mitochondrial uptake of Ca^2+^ is crucial to the regulation of their intrinsic functions. Ca^2+^ in mitochondria plays a significant role in regulating metabolic activity (Gherardi et al., 2020). The mitochondrial calcium transporter (MCU) is one of the most important Ca^2+^ transport complexes within mitochondrial Ca^2+^ uptake channels. A disturbance in mitochondrial Ca^2+^ may lead to impaired function of the MCU and abnormalities in mitochondrial metabolism and kinetics (Wu et al., 2021). Furthermore, mitochondrial Ca^2+^ overload can lead to the opening of the mitochondrial membrane permeability transformation pore (mPTP), swelling of mitochondria, and the production of ROS (Strubbe-Rivera et al., 2021). Researchers have reported that fatigue patients and aging mice have reduced mitochondrial Ca^2+^ uptake and muscle function (Debattisti et al., 2019; Yang et al., 2021). Therefore, mitochondrial Ca^2+^ uptake imbalances impair muscle fiber contraction, and restoring mitochondrial Ca^2+^ uptake can improve skeletal muscle function in aging mice (Debattisti et al., 2019; Yang et al., 2021).

Research has demonstrated that regular exercise promotes the repair and maintenance of Ca^2+^ release units, Ca^2+^ entry units, and mitochondria during denervation and aging (Protasi et al., 2021). Li compared the effects of HIIT and MICT on Ca^2+^ conduction in an aged rat model (Li FH. et al., 2019). According to the study, HIIT can increase the expression of some genes, thereby improving Ca^2+^ signal transduction and systolic function by supporting these proteins. Both HIIT and MICT improved Ca^2+^ signaling and muscle contractile protein expression in older skeletal muscles, which offset age-related changes in excitatory contraction coupling. Additionally, Zampieri examined mitochondrial Ca^2+^ homeostasis in response to neuromuscular electrical stimulation and proprioceptive training (Zampieri et al., 2016). Based on 9 weeks of training, both types of training can increase MCU protein levels, thus improving muscle function and structure. Under electron microscopy, ultrastructural analysis has revealed that mitochondrial changes in neuromuscular electrical stimulation training muscles may be mediated by increases in the expression of mitochondrial fusion protein OPA1. Furthermore, mitochondria that lack Drp1 have a larger morphology and are more dysfunctional. Therefore, mitochondrial dynamic balance is essential for maintaining mitochondrial Ca^2+^ homeostasis (Favaro et al., 2019). Exercise has been shown to maintain mitochondrial Ca^2+^ homeostasis in older skeletal muscle based on current evidence. Nevertheless, more research is required in the future to determine the optimal exercise form, intensity, and duration.

### Exercise ameliorates sarcopenia by inhibiting mitochondrial oxidative stress

Two fundamental biological processes determine the level of oxidative stress in the older adult with sarcopenia: the production of ROS and the decline in antioxidant defenses caused by aging (Ji, 2002). The imbalance between ROS production and antioxidant defense systems results in mitochondrial oxidative stress. Several pathways (e.g., electron transfer chain (ETC.) and aging can promote excessive ROS production, which can cause mitochondrial damage (Selivanov et al., 2011; Brand, 2016; Thoma et al., 2020). A lack of exercise also elevates ROS, which further reduces muscle function and mass (Waltz et al., 2018). It has been demonstrated that increased ROS is associated with fiber atrophy and necrosis in sarcopenia cases (Brioche and Lemoine-Morel, 2016). Therefore, high ROS levels in mitochondria contribute to the pathogenesis of sarcopenia in older adults. The mitochondrial antioxidant defense system includes catalase (CAT) and superoxide dismutase (SOD). A higher level of CAT is effective at reducing ROS production, prolonging lifespan, and reducing fatigue and atrophy in muscles (Selsby, 2011; Xu et al., 2021). Moreover, an imbalance between ROS and antioxidant defenses leads to high levels of oxidative stress that exacerbate ROS-induced sarcopenia (Sullivan-Gunn and Lewandowski, 2013). Therefore, inhibiting mitochondrial oxidative stress is crucial.

Exercise-induced ROS production improves mitochondrial efficiency, while chronic ROS production is detrimental to mitochondria. This may be because exercise can improve the coupling efficiency of, ETCs, reduce electron leakage and excessive mitochondrial ROS production, as well as restoring the balance of mitochondrial oxidative stress in the body to a healthy level (Corsetto et al., 2019). Furthermore, studies have shown that training time has a significant effect on CAT activity. An aerobic exercise can cause a transient peroxidation state in white adipose tissue, increasing the expression of antioxidant defenses (Matta et al., 2021). This may be due to the activation of the redox effector factor-1 (Ref1) and nuclear factor e2-related factor 2 (Nrf2) antioxidant defense pathways, which may prevent cellular oxidative stress resistance during acute exercise (Wang et al., 2016). Furthermore, chronic exercise can improve the antioxidant defense system. An 8-week aerobic and resistance exercise program increased SOD2 and CAT activity in aged rats (Vilela et al., 2018). Furthermore, 8-month HIIT and MICT can upregulate the expression of SOD2 and Acetaldehyde dehydrogenase (ALDH) (Li FH. et al., 2019). Nevertheless, the level of SOD2 in the MICT group did not reach statistical significance. Further, HIIT provides greater benefits to aged rats than MICT in terms of combating chronic low-level inflammation and oxidative stress (Li et al., 2018). A possible explanation for this may be related to the tissue-specific oxidative stress response of HIIT and MICT (Groussard et al., 2019). Therefore, acute aerobic exercise and chronic HIIT improve mitochondrial oxidative stress more effectively.

#### Exercise ameliorates sarcopenia by inhibiting MtDNA

Mitochondrial DNA replication, deletion, and mutation degrees increase with age, directly affecting cell metabolism and mitochondrial function (Cortopassi and Arnheim, 1990; Harper et al., 2021; Herbst et al., 2021). Additionally, mtDNA mutant mice showed a higher level of mitochondrial fission, autophagy, and mitochondrial oxidative stress (Joseph et al., 2013; Ma et al., 2020). As a result, in older adults with mitochondrial dysfunction, mutated mtDNA may result in reduced oxidative phosphorylation. The effect of this is to affect the assembly of functional, ETC., complexes. However, it does not increase oxidative stress, resulting in skeletal muscle apoptosis and sarcopenia in the long run (Hiona et al., 2010). In muscle fibers containing, ETC., defects, mtDNA deletion mutations increased by 1,200%, muscle fiber numbers decreased by 18%, and muscle mass loss worsened by 22% (Herbst et al., 2016). These results confirm the importance of mitochondrial DNA deletions and mutations in the development of muscle atrophy in older adults. This is in contrast to the reactions observed in normal aging muscles and may provide a means for detecting muscle atrophy at an early stage.

Exercise can regulate mtDNA levels in the skeletal muscles of the older adult. For example, exercise can promote mtDNA replication in older skeletal muscles. Upon reviewing the literature, Erlich found that exercise-induced mitochondrial biogenesis occurred simultaneously with an increase in mtDNA copy number (Erlich et al., 2016). Furthermore, exercise may stimulate the synthesis of mtDNA in human skeletal muscle (Robinson et al., 2011). Acute endurance exercise is capable of increasing P53 and mitochondrial DNA levels (Saleem and Hood, 2013). Therefore, exercise may be an effective mechanism for sarcopenia treatment by activating P53 to promote metabolic function. P53 can also interact with TFAM and DNA polymerase gamma to promote the binding of mtDNA and maintain mtDNA integrity (Achanta et al., 2005; Park et al., 2009; Park et al., 2016). Moreover, exercise can reduce mtDNA mutations in skeletal muscle and normalize protein levels (Maclaine et al., 2020). Safdar found that mtDNA in sedentary and exercised PolG mice contained similar numbers of mutations (Safdar et al., 2016). Nevertheless, sedentary mice suffer from more non-mutational damage, which can be mitigated through exercise. Additionally, the significant relief of mtDNA mutation phenotypes in muscle from exercise may not be due to a reduction in mutation load, but rather to a reduction in damage to mtDNA and/or oxidative stress. Therefore, endurance exercise promotes mtDNA replication and inhibits mtDNA mutations. Nevertheless, further investigation is required to identify the mechanisms of mitochondrial dysfunction and mtDNA dysfunction.

### Exercise ameliorates sarcopenia by inhibiting mitochondrial apoptosis

**FIGURE 1:**
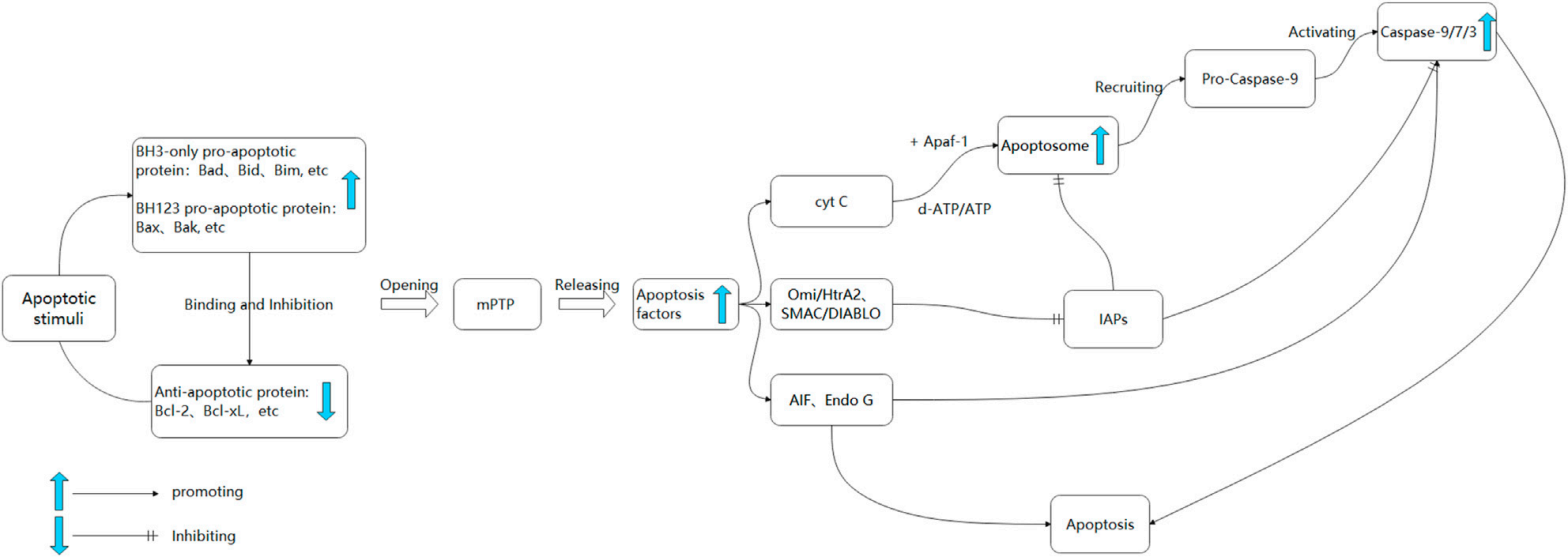
The mitochondrial apoptosis pathway. Upon exposure to apoptotic signals, the upregulation of pro-apoptotic proteins occurs, resulting in their binding to anti-apoptotic proteins. This interaction leads to an increase in mitochondrial permeability and the subsequent opening of the mPTP, ultimately resulting in the release of apoptotic factors (Cruz-Jentoft and Sayer, 2019). Cyt C binds to Apaf-1, forming a mitochondrial complex facilitated by ATP/dATP. This complex recruits Caspase 9 proenzyme, forming apoptosomes and initiating downstream Caspase cascade reactions (Wu et al., 2022). SMAC, Omi/HtrA2 can bind to IAPs, inhibiting Caspase 3 and 9 (Chen Z. et al., 2021). AIF and Endo G translocate to the nucleus, causing chromatin condensation and large-scale DNA fragmentation. In the cytoplasm, AIF activates Caspase 3 and subsequently activates Caspase 9.

Upon stimulation by internal apoptotic factors, such as oxidative stress, calcium overload, and DNA damage, an imbalance between anti-apoptotic proteins (e.g., B-cell lymphoma-2 (Bcl-2), Bcl-xL) and pro-apoptotic proteins (e.g., Bcl-2-associated X protein (Bax), Bak, Bid) occurs, increasing mitochondrial outer membrane permeability and opening of the mitochondrial permeability transition pore (mPTP) (Lindsay et al., 2011). This process leads to the release of mitochondrial pro-apoptotic factors, including cytochrome C (cyto C), apoptosis-inducing factor (AIF), the second mitochondria-derived activator of caspases/direct inhibitor of apoptosis-binding protein with low pI (SMAC/DIABLO), high-temperature requirement protein A2/omi (HTRA2/OMI), and endonuclease G (ENDOG), into the cytoplasm (Bano and Prehn, 2018). Upon being released into the cell, cyto C engages in an interaction with Apaf-1, resulting in the formation of an apoptotic complex that is facilitated by ATP and dATP (Yadav et al., 2020). This complex serves to recruit and activate Pro-Caspase9, ultimately giving rise to the Caspase9 holoenzyme. The entire Caspase9 enzyme then proceeds to activate Caspase3 and Caspase7, thereby initiating the caspase cascade reaction and ultimately culminating in cell apoptosis (Ola et al., 2011). The activation of Caspase3 and Caspase7 can be inhibited by Apoptosis inhibitory proteins (IAPs), which in turn can impede the process of cell apoptosis (Scott et al., 2005). The release of SMAC/DIABLO and HTRA2/OMI from mitochondria into the cell results in their binding to IAPs, thereby releasing the inhibitory effect of IAPs and indirectly promoting apoptosis (Scott et al., 2005; Vande Walle et al., 2008). Simultaneously, apoptosis can activate the E3 ubiquitin ligase MAFbx/atrogin-1 and Muscle RING finger 1 (MuRF1), leading to increased protein degradation and exacerbating aging-induced skeletal muscle loss (Cheema et al., 2015) (Figure 1). Consequently, the upregulation of pro-apoptotic processes may contribute to age-related muscle mass and cachexia loss.

Exercise training has been shown to reduce metabolic changes caused by mitochondrial apoptosis. As an example, exercise training at 60% VO_2_ max for 6 weeks can reverse age-related catabolism and apoptosis (Ziaaldini et al., 2015). Additionally, treadmill exercise training for 12 weeks decreased Caspase-3, Bax, and Bax/Bcl-2 ratios, as well as increased anti-apoptotic Bcl-2 in the white gastrocnemius and soleus muscles of aged rats (Song et al., 2006). The activity of nuclear factor kappa B (NF-B) increases with exercise training and decreases with aging (Song et al., 2006). Based on these results, aerobic exercise may be able to reduce fiber atrophy and proapoptotic signal transduction in aging skeletal muscle. Compared to the effects of not exercising during adulthood or starting regular exercise later in life, lifelong exercise in rats increases autophagic activity and prevents skeletal muscle apoptosis (Gao et al., 2021). Additionally, Zeng believes that both aerobic exercise and resistance exercise can inhibit excessive apoptosis caused by the upregulation of Bcl-2 and downregulation of Bax in the skeletal muscles of aged rats (Zeng et al., 2020). However, resistance exercise has a greater impact on anti-apoptosis (Zeng et al., 2020). It may be possible that resistance exercise increases autophagic activity and reduces apoptosis in older adult skeletal muscle by regulating insulin-like growth factor-1 (IGF-1) and its receptors, and downregulating Akt/mTOR and Akt/FOXO3a signaling pathways (Luo et al., 2013). Alternatively, resistance exercise stimulation of autophagy may prevent NOD-like receptor thermal protein domain associated protein 3 (NLRP3) activation and reduce apoptosis in peripheral blood mononuclear cells (PBMCs) of the older adult (Mejías-Peña et al., 2017). To compare the effects of HIIT and resistance exercise, Su conducted a 32-week intervention and found that both showed a decrease in mitochondrial apoptosis (Su et al., 2022). Resistance training has a greater effect on reversing age-related muscle loss than HIIT. In contrast, HIIT is more effective in suppressing proapoptotic factors over the long term (Su et al., 2022). However, Further research is required to validate the potential of HIIT in enhancing sarcopenia among older adults through the inhibition of mitochondrial apoptosis.

## Conclusions and perspectives

**FIGURE 2:**
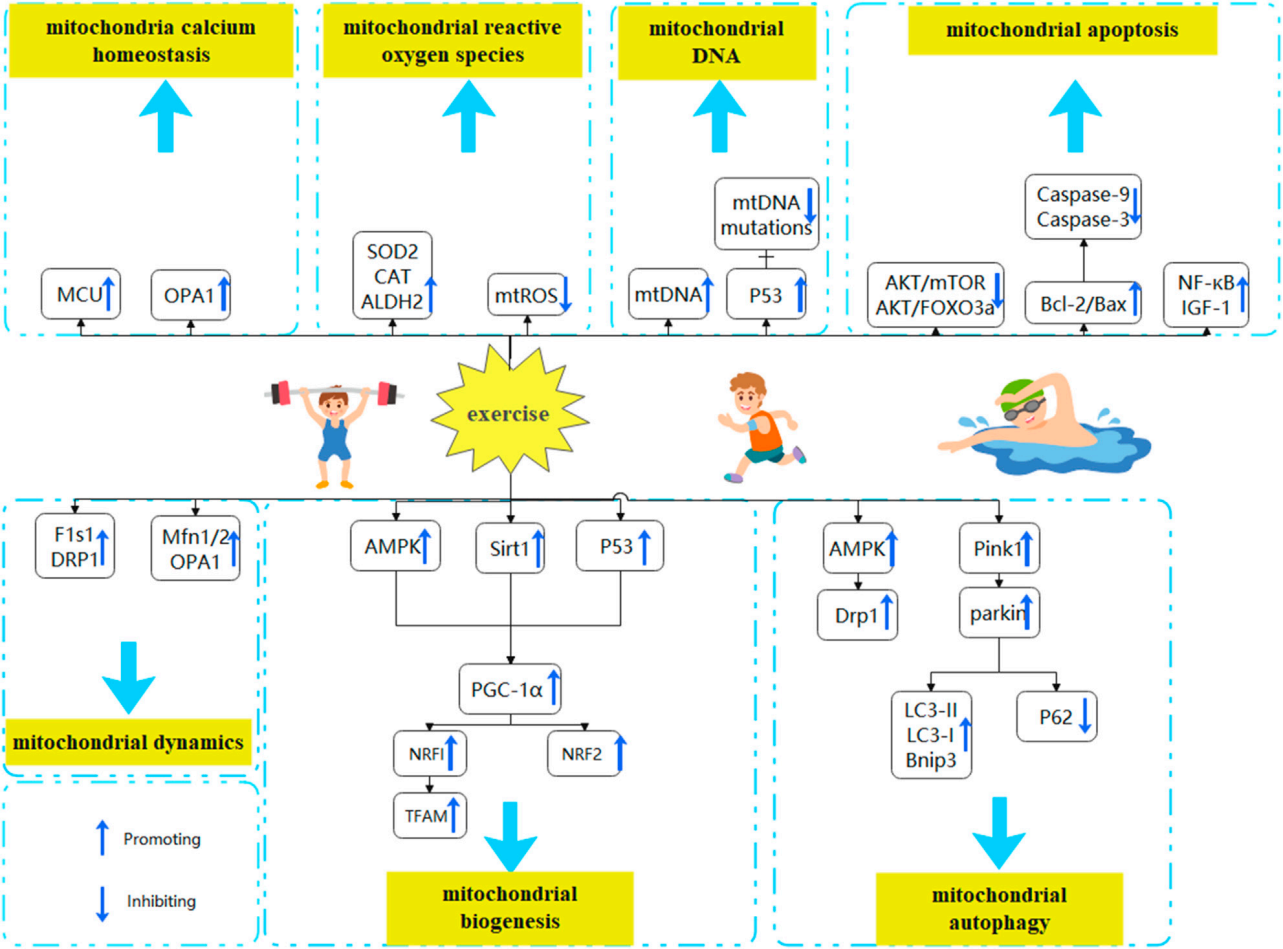
Exercise to improve mitochondrial dysfunction in the treatment of sarcopenia. Mitochondrial dysfunction is characterized by alterations in mitochondrial biogenesis, mitochondrial dynamics, mitochondrial autophagy, mtDNA, mitochondrial calcium homeostasis, mitochondrial oxidative stress, and mitochondrial apoptosis. Exercise may be able to reverse the aging process of skeletal muscle by modulating mitochondrial dysfunction **(A)** Exercise promotes mitochondrial biogenesis. By activating the AMPK, P53, and SIRT1 pathways and promoting PGC-1α expression, exercise reverses aging effects **(B)**Exercising balances mitochondrial dynamics. By increasing the expression of Mfn1/2, OPA1, DRP1, and FIS1, exercise reverses aging effects **(C)** Exercise enhances mitochondrial autophagy. By increasing the expression of AMPK and PINK1/Parkin pathways, promoting LC3-II/LC3-I ratio and BNIP3 expression, and downregulating p62 expression, exercise reverses aging effects **(D)** Exercise maintains mitochondrial Ca^2+^ homeostasis. By promoting MCU and OPA1 expression and restoring mitochondrial Ca^2+^ uptake, exercise reverses aging effects **(E)** Exercise reduces mitochondrial oxidative stress. By inhibiting mitochondrial ROS synthesis and promoting the production of antioxidant defense systems (CAT, SOD2, ALDH2), exercise reverses aging effects **(F)** Exercise reverses aging effects by promoting mitochondrial DNA synthesis and replication through P53 and inhibiting mtDNA mutations **(H)** Exercise decreases mitochondrial apoptosis. By downregulating Akt/mTOR and Akt/FOXO3a signaling pathways, and upregulating IGF-1, NF-κB, and Bcl-2/Bax ratios, exercise reverses aging effects.

With aging and inactivity, mitochondrial dysfunction, muscle reduction, or even muscle atrophy occur within the skeletal muscles of older adults and animals. This review analyzes the effects of exercise on mitochondrial biogenesis, mitochondrial dynamics, mitochondrial autophagy, mtDNA, mitochondrial Ca^2+^ homeostasis, mitochondrial oxidative stress, and mitochondrial apoptosis in aged rats and adults with sarcopenia. This review also sheds light on the interaction and potential mechanisms of mitochondrial dysfunction following exercise (Figure 2). Furthermore, this review summarizes the best exercise forms, intensities, and timings for regulating mitochondrial dysfunction. There is evidence that exercise improves the mitochondrial function of older adults with Sarcopenia. However, exercise form, intensity, and duration are significant factors that affect exercise effectiveness. HIIT has been shown to effectively stimulate mitochondrial biogenesis and dynamics in aged rats, thereby facilitating the generation of new mitochondria or the restoration of dysfunctional ones. Moreover, it has been observed to mitigate oxidative stress and mitochondrial apoptosis in aged rats. Moderate-intensity exercise maintains mitochondrial dynamic balance, promotes mitochondrial autophagy, reduces mitochondrial DNA damage and apoptosis, and eliminates dysfunctional mitochondria from the body. Acute exercise can promote mitochondrial fission and reduce mitochondrial oxidative stress. Chronic exercise is more suitable for improving mitochondrial dysfunction in older sarcopenia patients. Therefore, Moderate-intensity exercise over 4 weeks potentially mitigates sarcopenia in older adults by ameliorating mitochondrial dysfunction. HIIT has demonstrated potential as a viable approach to addressing sarcopenia in aged rats. However, further investigation is required to validate its efficacy in treating sarcopenia in older adults.
